# Remote Monitoring of Chronic Diseases: A Landscape Assessment of Policies in Four European Countries

**DOI:** 10.1371/journal.pone.0155738

**Published:** 2016-05-19

**Authors:** Katherine Rojahn, Suzanne Laplante, James Sloand, Claire Main, Aftab Ibrahim, Janet Wild, Nicky Sturt, Thelga Areteou, K. Ian Johnson

**Affiliations:** 1 Baxter Healthcare Corporation, Deerfield, IL, United States of America; 2 Baxter Healthcare Ltd, Compton, United Kingdom; 3 Double Helix, London, United Kingdom; Postgraduate Medical Institute, INDIA

## Abstract

**Background:**

Remote monitoring (RM) is defined as the surveillance of device-transmitted outpatient data. RM is expected to enable better management of chronic diseases. The objective of this research was to identify public policies concerning RM in four European countries.

**Methods:**

Searches of the medical literature, the Internet, and Ministry of Health websites for the United Kingdom (UK), Germany, Italy, and Spain were performed in order to identify RM policies for chronic diseases, including end stage renal disease (ESRD), chronic pulmonary obstructive disease (COPD), diabetes, heart failure, and hypertension. Searches were first performed in Q1 2014 and updated in Q4 2015. In addition, in depth interviews were conducted with payers/policymakers in each country. Information was obtained on existing policies, disease areas and RM services covered and level of reimbursement, other incentives such as quality indicators, past/current assessments of RM technologies, diseases perceived to benefit most from RM, and concerns about RM.

**Results:**

Policies on RM and/or telemedicine were identified in all four countries. Pilot projects (mostly in diabetes, COPD, and/or heart failure) existed or were planned in most countries. Perceived value of RM was moderate to high, with the highest rating given for heart failure. Interviewees expressed concerns about sharing of medical information, and the need for capital investment. Patients recently discharged from hospital, and patients living remotely, or with serious and/or complicated diseases, were believed to be the most likely to benefit from RM. Formal reimbursement is scarce, but more commonly available for patients with heart failure.

**Conclusions:**

In the four European countries surveyed, RM has attracted considerable interest for its potential to increase the efficiency of healthcare for chronic diseases. Although rare at this moment, incentives to use RM technology are likely to increase in the near future as the body of evidence of clinical and/or economic benefit grows.

## Introduction

The terms “telemedicine” and “telehealth” are used to describe the exchange of medical information electronically between one site and another with the aim of improving the health of patients.[1] There is no universal definition for these terms, and they are sometimes used interchangeably.[1, 2] These terms cover a wide range of electronic health (eHealth) services and technologies, including remote monitoring (RM) of a patient’s clinical status, teleconsultations, tele-education, tele-diagnostics and tele-laboratory, and may involve videoconferencing and/or transmission of still images.[2] RM is a technology that enables monitoring of patients beyond conventional clinical settings, for example, in the home. A device or software is used to collect a patient’s clinical data, such as blood pressure, blood glucose concentration, temperature, or body weight, and the data are then transmitted to a hospital, physician’s office, clinic, telemedicine vendor, or other party, for monitoring and analysis. On the basis of the RM data received, an appropriate healthcare professional may decide to initiate a preventative therapeutic intervention or change the patient’s prescription.

Incorporating RM into chronic disease management programmes can significantly improve an individual’s quality of life, allowing patients to maintain independence, prevent complications, and minimise costs.[3–5] This is particularly important when patients are managing self-care processes such as home dialysis.[6, 7] By RM and trend analysis of physiological parameters, the technology enables early detection of deterioration of non-compliance and a patient’s clinical condition. There is increasing evidence that RM data can help physicians and nurses to proactively manage a patient’s treatment. RM has demonstrated some clinical benefit for patients with chronic disease, including reduced all-cause or disease-specific hospitalisations and emergency visits for patients with diabetes, end stage renal disease (ESRD), heart failure, chronic pulmonary obstructive disease (COPD), and hypertension.[8–12] RM has also been associated with reduction in mortality in patients with heart failure, COPD, and diabetes.[10, 11, 13, 14] However, the effectiveness of RM, and other telemedicine services, may vary widely according to different factors related to the patient, their health condition, the endpoints measured, the quality of the data collection, and the healthcare system in which the intervention is being used. For example, a recent systematic review of the effectiveness, costs, and acceptability of telemedicine found that the effect on all-cause hospital admissions (at median eight months follow-up) ranged from a decrease of 64% to an increase of 60%, across the 11 heart failure studies that were included.[3] On the other hand, a recent meta-analysis showed that patients on RM had a 29% lower rate of heart failure-related hospitalisations (RR = 0.71; 95%CI: 0.60–0.83) and a 20% lower rate of all-cause mortality (RR = 0.80; 95%CI: 0.68–0.94).[8]

Although, currently, there is only limited evidence of clinical benefit in patients with ESRD, it is believed that RM might enable better management of patients receiving haemodialysis or peritoneal dialysis in a home setting.[6, 7, 14] Thus, RM has the potential to increase access to care and decrease healthcare delivery costs.[14] However, RM is highly dependent on patients’ ability to use the technologies involved, with additional barriers, such as cost, preventing its widespread use.[2, 15] There is currently little evidence that RM represents an efficient use of healthcare resources. Hence, healthcare systems have not yet put in place reimbursement guidelines or proper funding for RM services. This may deter their adoption into clinical practice, as healthcare providers would have to assume the costs.[2, 16]

The objective of this study was, therefore, to understand the current policies for the support and reimbursement of RM for home-based therapies, particularly for chronic disease states, in four European countries: UK, Germany, Italy, and Spain.

## Materials and Methods

### Research objectives

Specific objectives of the research included: 1) to understand the current public policies for RM in UK, Germany, Italy, and Spain; 2) to identify which countries are investing in RM programmes and have centres of excellence that use RM devices; 3) to identify which countries have implemented incentives and policies to support RM for home-based therapies; and 4) to identify and understand the barriers and, in particular, the perceived evidence gap in those that have not yet implemented such incentives and policies.

### Research methods

Two approaches were used to answer the research question: desk research involving medical literature and internet searches, and primary research involving one-hour in depth interviews with payers, policymakers, and clinicians.

To identify RM policies for chronic diseases, electronic searches of the published medical literature were conducted on MEDLINE, Embase^®^, Embase^®^ Alert, and the Cochrane Library, and wider Internet searches were also performed, using the Google search engine. Search terms for both the medical literature and internet searches included ‘telemedicine’ and related terms, as well as ‘policy’. Citations were selected for full review if they met specific inclusion criteria (e.g. specific country, policy, guidelines). Selected citations were reviewed and the following information was extracted: type of services covered, reimbursement level, incentives (e.g. quality indicators), past or ongoing assessments of RM technologies, disease areas perceived to benefit the most from RM, and concerns about RM use or implementation.

Several different methods of searching the Internet were employed to identify documents relating to telemedicine/RM: structured searching of public electronic databases; targeted key word and key phrase searching of previously identified key websites and standard internet search engines e.g. Google; following links from key websites to other websites of interest, or to locate relevant documents. In order to obtain an appropriate level of coverage, searches were conducted from more than one perspective, e.g. following threads relating to the type of document, the specific therapy area, intervention and management. All searches were originally performed in February 2014 and updated in October 2015.

Additional primary research was conducted through one-hour in depth interviews with a sample of three national policy makers/payers (1 from the UK, 1 from Germany, 1 from Spain), ten regional payers (3 from the UK, 3 from Germany, 1 from Italy, 3 from Spain), and six clinical experts with experience in developing RM initiatives (1 from the UK, 1 from Germany, 3 from Italy, 1 from Spain). Four experienced native interviewers were involved, one for each country.

The interviews were conducted in accordance with the ethical and legal guidelines produced by the European Pharmaceutical Market Research Association (EphMRA) in order to protect the rights of the consenting respondents, maintain confidentiality and anonymity, and avoid promotion. Interviewee questions were divided up into four sections: Section 1 requested information on the interviewee’s basic professional background, and their relevant experience with the reimbursement of medical devices; Section 2 focused on country/region specific questions on RM reimbursement, including funding, evidence requirements, and who influenced the adoption of policies; Section 3 asked about the current and future RM reimbursement landscape, such as the information that is required to drive reimbursement adoption, the diseases RM is most suitable for, and the type of clinical or health economics evidence necessary to obtain reimbursement. Finally, Section 4 questioned the interviewees on RM reimbursement models and incentives for physicians and providers, and the key drivers and barriers to RM adoption (a sample interview discussion guide is provided in the online Supporting Information).

To minimize risk of bias, the interviewer remained neutral in tone and language, and the questions asked were simple and clear, to reduce risk of misunderstanding. The questions were neutral in approach (leading questions were avoided), and all interviewees were asked the same questions. The breadth of roles and countries from which the interviewees were recruited also helped to reduce bias.

Transcripts of the interviews were produced, and these were then coded and analysed by country for recurrent themes and ideas that were echoed by all professionals. Similarly, any items that were distinctly highlighted as being different to the general consensus were noted. This analysis was then performed by a second reviewer, to assess inter-rater reliability.

## Results

### Policies and initiatives on RM and/or telemedicine: findings from the desk research)

Overall, there was a lack of alignment on definitions, research activities, and regional or national adoption of RM. Policies and initiatives on RM specifically and/or telemedicine in general were identified in all countries studied (Table 1) and most had regional or national pilot projects underway (these were mainly in diabetes, heart failure, or COPD).

**Table 1:** Policies and initiatives on RM and/or telemedicine in the UK, Germany, Italy, and Spain.

**UK[2, 11–13, 16, 21–23]**	• Whole Systems Demonstrator Project (WSD): a large randomised controlled trial conducted to justify public funding for telehealth services in England
	• 3millionlives Campaign: aimed to expand telemedicine, mobile and telecare access to 3 million individuals in England with long-term conditions by 2017; superseded by the Technology Enabled Care Services (TECS) programme at NHS England
	• Remote Care Monitoring Preparation Scheme: £0.21 per patient payment to GP practices in 2013/14 for preparation for introduction of Remote Care Monitoring Directly Enhanced Service (DES) in 2014/15; DES ended March 2014
	• All-Wales Telemedicine Development Programme: three demonstrator projects in Wales to test a sustainable service model to manage and treat chronic diseases through telecare and telemedicine
	• NHS England A Call to Action: Commissioning for Prevention
**Germany[21, 22, 24]**	• Although no EBM codes exist for RM/telemedicine, a ‘criteria catalogue’ for telemedicine (established in 2013) provides guidance on what telemedicine studies should include to obtain an EBM code
	• Strong interest in telemedicine and e-Health in Germany has led to capital investment being made available to fund large scale clinical trials to better define the clinical benefits of RM and telemedicine and drive telemedicine projects
	• Some regions (e.g. Bayern, Baden-Württemberg and Wiesbaden) have negotiated integrated care contracts between the health insurer and the provider
	• Telemedicine strategies in Bayern, Nürnberg, and Sachsen-Anhalt may lead to future RM technologies; these strategies have helped to launch RM programmes such as *Diabetiva* in Sachsen-Anhalt
	• Germany’s developed IT health infrastructure (e.g. electronic health card) may be able to offer RM and other telemedicine technologies in the future but is currently only used for billing purposes
**Italy[20, 25–29]**	• National Observatory for the Evaluation and the monitoring of eCare Networks: an organisation initiated by the Ministry of Health in 2007, in agreement with the region of Emilia Romagna; created a web-platform where 700+ telemedicine initiatives are self-reported on the basis of the technology, organisation, cost, and clinical value; developed “Guidelines for the Development of Telemedicine Best Practices”
	• RM pilot projects are implemented at a regional level by the *Azienda Sanitaria Locale* (ASLs), the regional health authority
	• Horizon Scanning: initiated by AGENAS (National Agency for Regional Healthcare), this programme evaluates emerging technologies including telemonitoring
	• Board of Health Technical table (*Tavolo Tecnico*): established in February 2011 to determine a strategic framework for telemedicine and define taxonomies/classifications
**Spain[30–34]**	• In addition to a national HTA (Institute of Carlos III), regional HTAs are also involved in the evaluation of RM technologies; the Research Unit for Telemedicine and Information Society (UITeS), a division under the national HTA, is attempting to address the standards issue and encourage uptake of RM/telemedicine
	• Platform of Innovation in Telehealth Systems (PITES) is a government-supported initiative that provides services and tools to support research groups (public, private and organisations) in obtaining evidence for new RM and telemedicine models that provide health care for chronic illnesses and dependency
	• Multiple projects throughout Spain use PITES as its infrastructure (e.g. Hospital Universitario Virgen del Rocío, Hospital Universitario Ramón y Cajal)
	• Telemedicine programmes have long been implemented at certain regions (e.g. Valcronic teleHealth program, since 2011 in Valencia region, TELBIL program since 2013 in Basque Country) and local hospitals (e.g. Hospital Clinic in Barcelona since 1999), specifically in patients with chronic disease
	• Regions with high levels of telemedicine/RM include: Catalonia, Canary Islands (high level of telemedicine not RM), Basque Country, Valencia Region and Andalucia; national technological standards may be an issue since interoperability varies between and within regions (e.g. Catalonia providers use different information systems, Valencia, Andalucía and Basque Country each have its own system)

The UK government has invested heavily in experimental RM activity for chronic heart failure, diabetes, and COPD. The Whole Systems Demonstrator Project (WSD) was the largest randomised controlled trial of RM conducted to date, and broadly aimed to evaluate the effectiveness and cost-effectiveness of telemedicine in people with chronic health conditions and social care needs.[17] The UK Department of Health report of ‘Headline Findings’ from the WSD study (2011) showed substantial reductions in mortality and healthcare resource utilisation,[18] but the study had mixed objectives, RM technologies, patient populations, and disease states, and subsequent reports have revealed results that are contradictory and ambiguous. Although RM is perceived to be suitable for patients with any chronic disease, it is unclear when it will be fully embraced by general practitioners (GPs) or specialists due to the lack of robust evidence of cost-effectiveness. The UK National Health Service (NHS) is actively looking for different ways of delivering care, and telemedicine/RM is a nationally recognised priority, but no national reimbursement mechanism currently exists.

In Germany, telemedicine (the broader category, which covers RM) has attracted strong interest from the national government. This has led to funding being made available for large-scale randomised clinical trials to drive telemedicine projects and better understand the benefits. Germany has one of the most technologically advanced healthcare infrastructures in Europe, having launched the e-health card (eGK) nationwide and developed a telematics infrastructure. However, hurdles in implementation have also hindered RM technologies due to software integration issues between physician’s offices and providers.

A number of telemedicine pilot studies have been initiated in Bayern, Nürnberg and Sachsen-Anhalt. The EBM (*Einheitlicher Bewertungsmaßstab*) is a reference list that regulates the compensation system of outpatient care in Germany, and no EBM codes currently exist for RM, but a ‘criteria catalogue’ has been established to provide guidance on what is required from telemedicine studies.

Following an initiative of the National Association of Statutory Health Insurance Physicians (KBV), from April 2016, a cardiac pacemaker check-up will be the first telemedicine procedure to be included in the EBM.[19] The KBV plans to introduce more remote procedures in the future in order to solve the problem of overfilled practices.

In Italy, RM pilot projects are implemented at a regional level by the regional health authorities (*Azienda Sanitaria Locale* [ASL]). ‘Mydoctor@home’ is a RM/telemedicine pilot in Piedmont that demonstrated positive outcomes and was approved for roll-out.[20]

As in the UK, there is no standardised national tariff for telemedicine in Italy, which is seen as a major barrier to adoption. However, the recently introduced Telemedicine National Guidelines (approved February 2014) aim to standardise the approach to telemedicine and propose a standardised tariff, which could lead to greater adoption of RM projects by ASLs.

In Spain, the recent economic downturn and financial constraints have resulted in a reluctance to fund the capital investment of RM initiatives. The majority of funding for RM projects is provided by international organisations (e.g., European Commission) or regional budgets. RM efforts have not been widely adopted at the national level; however, there are some regionally-driven RM initiatives. One such initiative is the Research Unit for Telemedicine and Information Society [UITeS] within the Institute of Carlos III, which develops standards alongside the initiatives of the Ministry of Health and Social Policy, and encourages uptake of RM/telemedicine. Autonomous Communities (ACs) of Basque Country, Andalucía, Catalonia, Valencia, Canarias, Madrid, and Navarra are seen as leaders in RM implementation in Spain due to their investment in healthcare technology. ACs can choose individually to fund RM initiatives and projects.

### Perceived value and potential benefit of RM: findings from the primary research)

**Fig 1:**
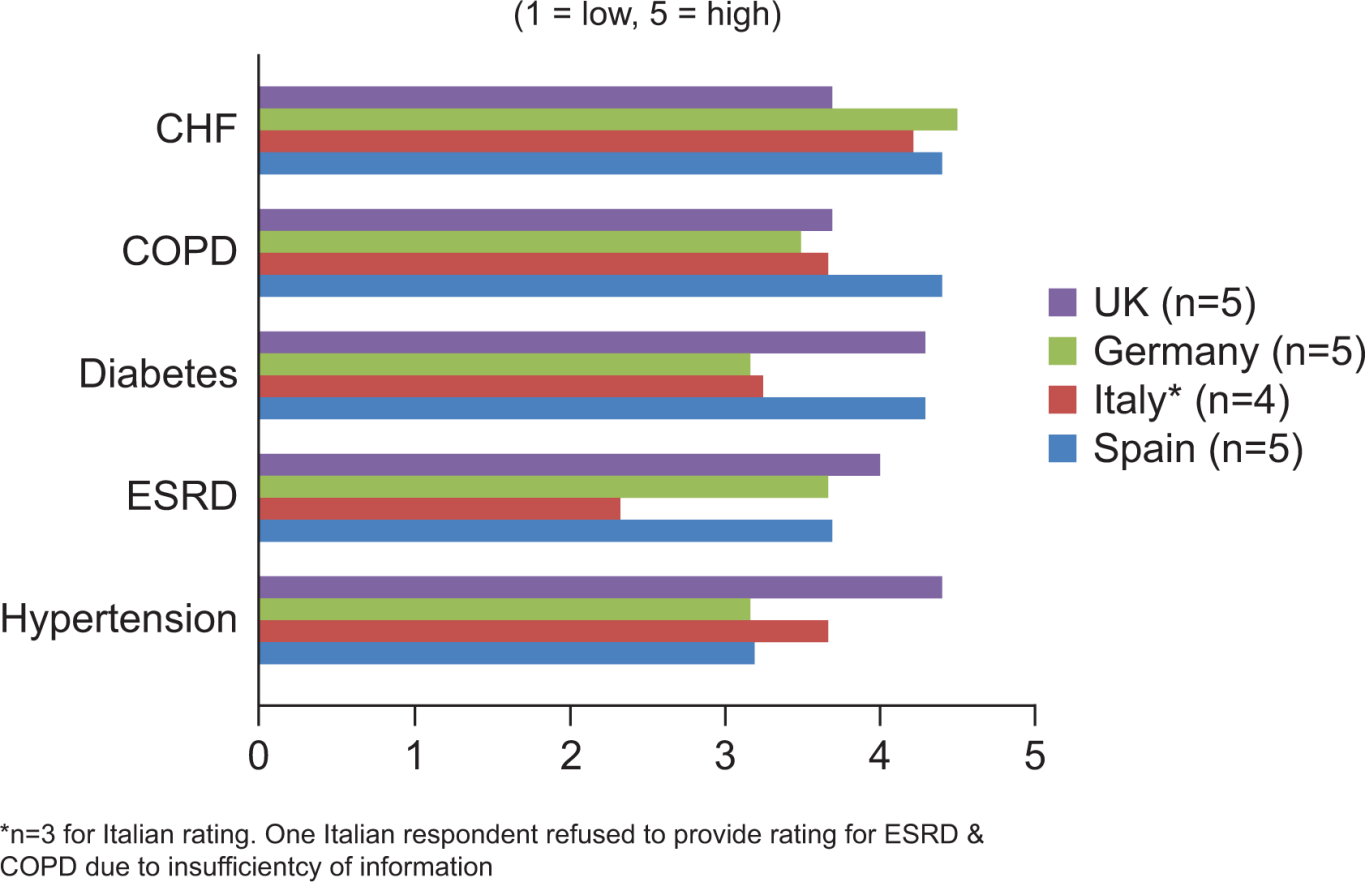
Potential for RM in UK, Germany, Italy and Spain, by chronic disease.

Experience of the application of RM in the community home setting is primarily limited to a few pilot studies in heart failure, hypertension, diabetes, and COPD. Amongst payers and policy makers in all of the countries surveyed, the value of RM was perceived to be moderate to high across all these chronic disease states, and also ESRD (Fig 1). Chronic heart failure (CHF) received the highest average rating (4.2±0.4 from a maximum of 5) whilst ESRD received the lowest (3.4±0.8 from a maximum of 5). In all countries studied, respondents indicated that CHF is a high priority area and that RM has high potential in this disease state.

This is consistent with the evidence in the literature of the benefits of RM for heart failure, and may be due to the high prevalence of heart failure in the population as well as its health economic burden. ESRD was not seen as a high priority for the application of RM, despite acknowledgments that it is associated with low quality of life for patients and high costs. There may also be a lack of awareness that approximately 30% of ESRD patients who initiate dialysis also have heart failure.[35] Accordingly, where respondents were unaware of RM projects in ESRD, its perceived potential was lower, and there was more awareness of the complexity of managing heart failure, hypertension, diabetes and COPD.

In the UK, chronic diseases in general were considered to be well suited for RM application. Hypertension scored highly, as there was a feeling from payers that this was a cardiovascular risk factor that could be managed better in the NHS. A regional payer also indicated that improved diabetes care was an area of interest that is growing, with particular emphasis on RM use in younger, type 1 diabetic patients. In addition, payers felt that RM applications in home dialysis could work for a cohort of stable patients. It was commented that ESRD is associated with high cost and low quality of life, and that treating patients in their own home could provide a better service.

In Germany, primary research findings indicated that all chronic conditions were suitable for RM. Heart failure was considered to have the highest potential due to the greatest clinical benefits RM could offer patients, and ESRD was rated as having lower potential. This could be due to payers being unaware of RM projects for ESRD patients. Diabetes and hypertension were considered to have the lowest RM potential because the evidence base for cost savings was poor and careful selection of eligible patients would be necessary. It was commented that it would not make sense to provide RM for a patient with diabetes whose treatment goals are always met perfectly, but rather to provide it for some 10% of patients who regularly miss their goals.

In Italy, heart failure was seen as having highest potential for RM projects, as it is a priority for ASLs and also attracts government interest. Hypertension and COPD were also thought to have high potential for RM, closely followed by diabetes. Overall, respondents in Italy gave ESRD the lowest rating for RM potential, as home dialysis is not so common in Italy, with 89.6% of dialysis taking place in an outpatient dialysis unit and only 10.4% in the home.[36] However, one payer suggested that RM initiatives in this area could encourage greater uptake of home dialysis.

In Spain, respondents indicated that COPD and chronic heart failure had highest RM potential and that they were high priority areas in their region. Hypertension was not seen as a priority for the application of RM devices, as other chronic diseases were deemed to be associated with greater morbidity. One payer felt the potential of RM for home dialysis was lower than other chronic diseases because they perceived the size of the affected patient population to be smaller. Mental illness and anticoagulation were also mentioned as areas with potential for application of RM.

In each of the countries studied, respondents indicated that they believed patients with rare, more serious or multiple diseases would benefit most from RM (Table 2).

**Table 2:** RM potential for patient population subsets, by country.

Criteria	UK	Germany	Italy	Spain
Patients suffering from rare, more serious, or multiple diseases	✓	✓	✓	✓
Patients immediately discharged from hospital, who require close monitoring/follow up	✓	✓		✓
Patients living in rural areas or without easy access to doctor/hospital	✓	✓		✓
Patients with frequent hospital readmissions	✓		✓	
Younger patients			✓	

In the UK, there was a strong indication towards patient stratification to identify patients who could benefit most. In addition to patients with more serious and multiple diseases, patients who have difficulty travelling to a nearby hospital, patients immediately discharged from hospital who require close monitoring follow-up, and patients with frequent hospital readmissions, were also cited as being likely to benefit from RM.

In Germany, respondents mentioned that patients in rural areas or where a doctor/hospital is not easily accessible are the most likely to benefit from RM. Patients suffering from rare diseases (e.g. congenital heart defects) and those immediately discharged from hospital were also considered to be key candidates for RM.

In Italy, both patients for whom hospitalisation can be avoided, and younger patients, were viewed as more eligible candidates for RM, while, in Spain, payers noted that RM would be strongly applicable to chronic disease patients with certain characteristics who require a very specific follow-up, those who cannot travel to hospital but who could be examined or tested remotely, and those with more serious diseases. There was a difference of opinion between respondents about whether or not RM is more appropriate for patients with a single chronic disease (stated by one respondent) or those with multiple co-morbidities (stated by other respondents).

### Reimbursement for RM: findings from both desk and primary research

Coverage and implementation of reimbursement for RM varied by country. In the UK, although there has been strong investment in telemedicine and RM from the NHS, a formal reimbursement mechanism (directly enhanced services [DES]) has recently been abolished.[12, 23] This scheme, the Remote Care Monitoring (Preparation) Scheme, made a payment of £0.21 (GBP) per registered patient available to participating GP practices.[12] This initiative was later removed due to lack of use, probably because the financial incentive was insufficient to cover the cost of the administrative work involved.[37]

Funding for RM is decided at a regional level by clinical commissioning groups (CCGs) in England, and other funding streams are available at a national level through NHS England initiatives to support out-of-hospital care. An example is “A Call to Action: Commissioning for Prevention”, which will fund RM projects commissioned by CCGs to help deliver different models of care.[11] In the UK primary research interviews it was commented that the “Call to Action” provides a good opportunity to engage with CCGs on RM projects, particularly if it offers the potential to reduce hospital activity. A number of other national initiatives have been driven by NHS England, aimed at promoting funding for RM. These include the ‘3millionlives’ campaign.[22]

This project aimed to provide benefit of RM/telemedicine to the estimated 3 million people with long-term conditions by 2017.[22] Corporate partners, such as Tunstall (the largest supplier of RM devices in UK), served on the Board of this plan.[38] The ‘3millionlives’ campaign has now been superseded by the Technology Enabled Care Services, which aims *“to help maximise the value of technology enabled care services for patients*, *carers*, *commissioners and the whole health economy*.*”* [16] Other RM partnership opportunities exist, with funding for innovative projects being coordinated by Academic Health Science Networks (AHSNs) between commissioners and providers as part of CCGs 5-year strategic plans.

In Germany, general funding or reimbursement of telemedicine is not currently available at a national level due to the lack of an EBM code. However, a ‘criteria catalogue’ was established in 2013 in an effort to define indications/limits and provide guidance on what telemedicine projects should include. In the absence of an EBM code, integrated care contracts (§ 140a SGB V) are the most frequently used funding model, and funding is available for RM initiatives in some regions (e.g. Bayern, Baden-Württemberg and Hessen) through integrated care contracts negotiated between individual health insurers (*Krankenkassen*), manufacturers, and physician organisations.[39] For national reimbursement to be considered, the clinical benefits of RM would need to be demonstrated to the Federal Joint Committee (*Gemeinsamer Bundesausschuß*, GBA) and the evaluation committee (*Bewertungsausschuß*) in order to obtain an EBM code and associated value.

In Italy, Telemedicine National Guidelines (established by the Ministry of Health in February 2014) present a greater opportunity for national RM reimbursement, which could lead to greater adoption of RM initiatives. The guidelines include principles on reimbursement and the economic assessment of telemedicine services. For reimbursement purposes, a standardised national tariff for telemedicine is proposed at a national level, which could also help fund RM projects. DRG codes are found in two regions of Italy (Lombardy has a DRG code for RM for chronic heart failure in the hospital setting only; Piedmont has a preliminary DRG that was expected to be formalised in 2015), but there is none at the national level. The lack of a standardised national tariff is a barrier to wider adoption of RM projects.

In Spain, there is no national reimbursement mechanism for RM given that health competencies are transferred to the ACs, and telemedicine is funded in various regions. For example, telemedicine for home haemodialysis (telenephrology) is being pioneered by two hospitals in Canarias in partnership with Canarian Health Service. Telenephrology does not currently include RM, but because there is a Canarias policy in place for telenephrology, reimbursement for RM may follow in the future. In addition, ACs, such as Catalonia, are exploring co-payments to alleviate funding shortages and are seeking to partner with stakeholders such as manufacturers to help drive the use of RM. In Spain, there is also a government-supported initiative to develop a technological platform to help research groups obtain evidence for RM and other forms of telemedicine that provide health care for chronic illnesses and dependency. This initiative, the Platform of Innovation in Telehealth Systems (PITES), provides services and tools for use by public and private research organisations.[30] Funding and support are provided through the Research Unit for Telemedicine and Information Society (UITeS), a division of Institute Carlos III.

Interviewee responses revealed differences between countries in the evidence (assessment criteria) on which they would prefer to base decisions on the adoption of RM and the populations that would derive most benefit (Table 3). In the UK, in addition to clinical and health economic data, patient feedback was an important factor for both clinical experts and payers. In Italy, respondents were interested in seeing cost-effectiveness data as well as cost-utility data for RM, with real life data on effectiveness, safety and patient compliance also being mentioned as having increasing value. In Germany, respondents were most interested in evidence showing an added benefit of RM in certain patient groups as well as a reduction in patient hospitalisation due to RM. In Spain, respondents were most interested in evidence of cost savings arising from RM, and the quality of the patient experience with an RM device. Some of the cost drivers that payers appeared to favour were RM programmes that could help with early disease diagnosis or disease analytics to stratify patients by risk of hospital admission before complications arise.

**Table 3:** Preferred evidence (assessment criteria) interviewees in UK, Germany, Italy, and Spain indicated they would like to see to support decisions on adoption of RM.

Criteria		UK	Germany	Italy	Spain
Clinical	Observational data comparing two cohorts of patients (e.g. comparing effectiveness and safety of RM versus usual care, or RM + standard of care versus standard of care alone)	✓	✓	✓	
Clinical	Positive patient mortality		✓		
Clinical	Real life data on effectiveness, safety and patient compliance			✓	
Health Economics	Cost-utility data	✓		✓	
Health Economics	Budget impact model			✓	
Health Economics	Cost per incident/intervention avoided	✓			✓
Health Economics	Cost-effectiveness data		✓	✓	
Health Economics	Cost-benefit analysis over time			✓	
Health Economics	Reduction in healthcare resource utilisation		✓		
Other	Evidence of patient satisfaction	✓			✓
Other	RM offers additional value (e.g. early diagnosis and analytics)				✓

Some countries are pursuing their own programmes to obtain clinical/economic evidence for the benefit of RM. For example, the UK Department of Health provided £31M for the 2008−2010 WSD study, which has not demonstrated clear cost-benefits to encourage wider RM uptake at the GP level.[40] There is activity for RM and peritoneal dialysis in Spain. The eNefro project in Seville to clinically and economically evaluate home RM for peritoneal dialysis is funded by Institute of Carlos III.

### Concerns, challenges and limitations—barriers to adoption of RM: findings from desk and primary research

During the interviews, payers often commented that RM would help to keep patients out of hospital, but changing standards of care is associated with many challenges (e.g. lack of alignment of incentives, misconceptions about the impact of RM on physician revenue, and the need for convincing clinical and economic data for RM).

Experience of RM in the community home setting is primarily limited to studies in heart failure, hypertension, diabetes, and COPD, and thus, despite acknowledgment of the benefit in patient populations with multiple comorbidities, and the presence of heart failure in the ESRD population, payers and policy makers were less convinced of the value of RM in ESRD patients.[35] The definitions and policies remain unclear due to early stage of RM/telemedicine technology implementation and infrastructural issues (e.g. interoperability of electronic medical record [EMR] systems, funding). Concerns about medical information sharing and the capital investment required were also prominent, while privacy, liability, and security were significant concerns expressed by the policy makers. Barriers to RM adoption identified in the four European countries studied are summarised in Table 4.

**Table 4:** Barriers to adoption of RM in four European countries, identified from findings from desk and primary research.

UK	• Lack of positive cost-effectiveness data has led to a weak value proposition for RM to Care Commissioning Groups (CCGs) and General Practitioners (GPs); CCGs are reluctant to fund the up-front capital investment for RM projects without proper cost-effectiveness evidence.
	• Resistance from physicians on the potential change in practice that may lead from RM (e.g. less face-to-face consultation, with perceived greater potential for patients to change GPs more easily).
	• Lack of integration of electronic medical records (EMRs) between primary and secondary care making sharing of patients’ records harder.
	• No national reimbursement mechanism currently exists for RM.
	• Disconnect between national government and local implementation by CCGs (leading to variable regional awareness of national RM campaigns).
Germany	• Lack of health economic and clinical benefit data on RM projects.
	• Large providers unwilling to invest in RM without robust clinical and economic evidence.
	• Krankenkassen (health insurers) that can invest in RM projects do not have the resources to evaluate them.
	• First version of the established criteria catalogue may be too general as guidance.
	• IT infrastructural issues (e.g., interoperability between physicians and hospitals, implementing eGK electronic health card system).
	• Physicians’ fear of losing revenue.
	• Data protection concerns may restrict implementation of RM.
Italy	• Lack of robust cost-effectiveness data.
	• Lack of a national standardised tariff for telemedicine hinders RM national uptake.
	• No regional DRG code or tariff established for RM in most regions.
	• Insufficient funds to make novel additions to current care provision.
	• Lack of central coordination to push RM at a national level.
Spain	• Economic downturn and financial constraints result in a reluctance to fund the capital investment of RM initiatives.
	• While the body of evidence grows, Autonomous Communities (ACs) are slowly, but steadily, adopting RM initiatives.
	• National technological standards remain an issue as interoperability varies between and within ACs (with providers using different EMR systems).
	• Regional differences in adoption, funding and implementation of RM projects.

## Discussion

Some common themes for all four of the European countries surveyed included the lack of clarity in definitions and policies in RM due to the early stage of implementation of RM/telemedicine technology, and that the application of RM in a home-based setting is currently limited to a few pilot studies in the areas of heart failure, diabetes, and COPD. Infrastructural issues, such as need for interoperability of EMR systems and lack of funding/reimbursement, were also commonly identified, and the need for robust clinical and cost-effectiveness data for RM was echoed across all four countries.

In the countries studied, RM appears to have considerable public support for its potential to increase healthcare efficiency in chronic diseases. However, there are inconsistent rates of RM implementation due to disconnect between national and regional decisions, and reimbursement/funding levels and incentives vary depending on the local adoption of RM and the depth of RM research. Incentives to use RM technology were found to be rare. Heart failure seems to be an area of greater awareness of the benefits of RM, both in terms of published studies and expert opinion, possibly due to the greater prevalence of this condition and its higher morbidity. Despite growing evidence for the use of RM in the management of patients with ESRD (including peritoneal dialysis and home haemodialysis),[6, 7, 14] this disease had the lowest recognition of RM value in this current research.

Efforts to develop technological standards are being made, although infrastructural IT challenges are currently a major barrier to wider adoption of RM in a number of European countries. Also, despite strong regional and/or national imperatives that are supporting the use of RM, the lack of standardised national tariffs may be hindering the introduction of RM. However, budgetary restrictions can be overcome by exploring opportunities with innovative funding streams, such as the involvement of corporate partners (for example, Tunstall, major suppliers of RM devices, supported the ‘3millionlives’ campaign in the UK),[38] and the potential to offset costs of RM through savings in service delivery.

Increasing healthcare expenses will continue to drive a need for more affordable options and there is evidence that use of RM can decrease the cost of healthcare delivery. Moreover, the time saved as a result of RM implementation could increase efficiency, and would allow healthcare providers to allocate more time to remotely educate and communicate with patients. The ‘Virtual Ward’ healthcare model is an example from the UK that uses an approach of risk stratification and integrated care, and demonstrated a reduction in costs, elective hospital admissions and outpatient attendance.[18, 41] In this model, originally described by Lewis in 2006,[42] each virtual ward is linked to a group of GP practices, and a patient is offered ‘admission’ to a ward if he/she is predicted to be at high risk of a future unplanned hospital admission. The patient remains in the community during their time on the virtual ward, and receives health and social care from a multidisciplinary team, which may be delivered by telephone, in the patient’s home, and/or at a local clinic.[41, 42] Due to its home based and virtual nature, virtual wards may be a suitable setting for piloting and launch of RM technologies.

Collaboration with appropriate local, regional or national partners is likely to be of value in the development of RM initiatives. For example, in the UK, the AHSNs have the potential to transform health and healthcare by putting innovation at the heart of the NHS. Acting as the key link between commissioners, providers, local authorities and industry, AHSNs have access to funding mechanisms to help explore the benefits of projects like RM initiatives, particularly where there are potential cost-saving benefits in delivering care closer to patients. AHSNs are also likely to collaborate with CCGs on RM initiatives where there is the potential to reduce the reliance on hospitals and the more costly care they provide.

In summary, in the four European countries surveyed, RM has attracted considerable interest for its potential to increase the efficiency of healthcare for chronic diseases. However, the rates of implementation have been inconsistent due largely to a lack of alignment between national and regional decisions and challenges around varying RM technologies. A lack of standardised national tariffs may be hindering the introduction and more widespread use of RM, although there are examples of RM adoption within each country, presumably with existing budgets.

The value of RM is multifaceted (e.g. patient outcomes, provider efficiency, societal impact) and, as such, represents a measurement challenged. Although telemedicine programs were initiated several decades ago and have experienced rapid growth ever since, there is a lack of reliable, comparative economic data for policy makers, program administrators, and other stakeholders. Most of the economic evaluations of telemedicine focus on program/technology cost estimates alone, and the full range of economic benefits of telemedicine programs is rarely considered and quantified, making it difficult for decision makers to compare different programs and to make an informed decision as to which are worth implementing from a societal perspective.[38, 43] Incentives, whether financial or otherwise, are likely to emerge as the body of evidence demonstrating the clinical, time- and cost-saving benefits of RM grows. It will also be important to explore existing programs for chronic disease management that may benefit from including RM, and new funding opportunities, particularly for RM, at the regional and national levels.

The main limitation of this study is that it focused specifically on RM. Whilst one might expect that the perceived value and hurdles would be the same for telemedicine in general, a study focusing on the broader elements of telemedicine and/or telehealth would need to be conducted to identify similarities and validate our findings.

## Supporting Information

S1 File Sample interview discussion guide (Germany). (DOCX)
