# Supplementary material for: Remote Monitoring of Chronic Diseases: A Landscape Assessment of Policies in Four European Countries
Source: PLoS One. 2016 May 19;11(5):e0155738. doi: 10.1371/journal.pone.0155738 (PMC4873167; doi:10.1371/journal.pone.0155738)
Supplement: S1 File — (DOCX) [file pone.0155738.s001.docx]

**Interview Discussion Guide- Germany**

**INTERVIEW QUESTIONS**

**Section 1 – Interviewee Background**

1. Please tell us about your background and your relevant experience with the reimbursement of medical devices, particularly remote monitoring technologies.

**Section 2 – Country/Region-Specific Questions on RM Reimbursement**

1. **[Germany]** Could you please describe the different ways that RM is currently funded? (e.g., integrate care contracts, statutory health insurances [KKs], Federal Joint Committee [GBA], hospital funded, private grants, etc.) *[Probe on reimbursement pathway]*
2. **[Germany]** Are you familiar with integrate care contracts? Can you please provide examples? Who were the involved parties (KK, device manufacturer, physician’s association, patient group)?
   1. How effective are the integrate care contracts in creating a pathway for remote monitoring programs to take place?
   2. What is typically the goal and outcome of the integrate care contracts?
   3. Do you believe that integrate care contracts will act as a framework for more permanent RM reimbursement policies to take place? Why or why not?
3. **[Germany]** Which KK currently has the most advanced RM policies? *[Probe on Bavaria and Nuremberg]*
4. **[Germany]** Which KK’s healthcare policies would be the most conducive to the successful adoption of RM?
   1. How do you think aforementioned policies compare to other KK’s?
   2. How would that KK’s future policy on RM would influence other KK’s?
5. **[Germany]** Why is RM activity concentrated at the regional level and not at the national level?
6. **[Germany]** How do reimbursement levels differ for RM
   1. By KK?
   2. By the recipient of data (e.g., physician’s office vs. hospital vs. a 3^rd^ party telehealth vendor or other)?
   3. By geographic region (e.g., rural vs urban)?
   4. By connectivity to a medical record system? Is it a prerequisite to RM?
7. **[Germany]** What role would IQWiG and comparative-effectiveness research (CER) play in the evaluation of an RM technology?
8. **[Germany]** Which stakeholder/s has been most instrumental to influencing the creation and the successful adoption of RM reimbursement policies? (KK’s, GBA, patients, physicians, hospitals, device manufacturers, pharma industry)

**Section 3 – Current and Future RM Reimbursement Landscape**

1. What would be the key criteria used to determine RM reimbursement coverage be? (e.g., budget impact model, cost-effectiveness data, technological platform, interconnectivity with EMR, ease of use by patients and providers, or none of the above: payers are agnostic)
   1. Would patient access to his/her own information for the self-management of the disease a consideration in reimbursement?
2. What clinical or demographic factors would be used to separate patients into different risk categories that determine how appropriate they are for RM technology? How do those factors affect reimbursement rates?
3. Would an RM device be able to pursue additional reimbursement if it had the data analytics functionality to provide clinical recommendations and intervene early in the disease management, or change the patient’s prescription?
4. In your opinion, who are currently the top RM companies? *[Answer could include device manufacturer, telecom company, or data company]*
5. In your opinion, is there a chronic disease that RM is most suited for? Please rate on a scale of 1 to 5 (5 having the greatest potential for RM, and 1 the least potential for RM) and provide rationale:
   1. Heart Failure
   2. Hypertension
   3. Diabetes
   4. COPD
   5. Chronic Renal Disease
   6. Other

*For each disease state, probe on:*

1. In your opinion, which patient characteristics benefit the most from RM (e.g., age, geography, comorbidities)? Why? Do you believe that reimbursement should be adjusted according to the patient characteristics?
2. Are you aware of any pilot RM projects or integrated care contracts for this chronic disease? How is it funded? What was the outcome of this pilot? *[Probe on: clinical, economic, and patient quality of life outcomes]*
3. In your opinion, why don’t you think that more publications have resulted from pilots? How is the evidence from pilots or integrated care contracts shared or utilized to push for reimbursement or stronger adoption?
4. In your opinion, who are the top RM device manufacturers for this chronic disease state? And why?
5. What type of clinical or health economics evidence would be necessary for a RM device to obtain reimbursement? And why?
6. How far should a payer and/or clinician’s perception of RM change for it to be widely covered? How can this best be addressed?
7. How are current integrated care contracts/pilots addressing the clinical data requirements, changes, or policies required?
8. How do you foresee the future landscape of RM changing in the next year? Within the next 5 years?

**Section 4 – RM Reimbursement Models and Incentives**

1. Of the RM devices that you are aware of, do those devices collect clinical data for one chronic disease or do they address multiple disease states?
   1. Will an RM device that collects data for only one chronic disease be expected to have greater pricing and reimbursement potential rather than a RM device that collects data for multiple chronic diseases?
   2. Which of the above two RM device models would be expected to have more success in the short and long term?
2. What do you think of a bundled reimbursement approach (one fee for device and monitoring/analysis service) as opposed to a separate reimbursement approach (separate fee for device and separate fee for monitoring/analysis service)? Which would you prefer?
3. How do you envision RM reimbursement payment schedules would work in the future? Daily/monthly/per patient at a flat/variable fee?
4. What types of physician or provider incentives are in place to support the adoption and/or use of RM?
5. What are the key drivers and barriers to RM adoption?
